# Regulation of Monocytes/Macrophages by the Renin–Angiotensin System in Diabetic Nephropathy: State of the Art and Results of a Pilot Study

**DOI:** 10.3390/ijms22116009

**Published:** 2021-06-02

**Authors:** Claudine Moratal, Audrey Laurain, Mourad Naïmi, Thibault Florin, Vincent Esnault, Jaap G. Neels, Nicolas Chevalier, Giulia Chinetti, Guillaume Favre

**Affiliations:** 1 Université Côte d’Azur, INSERM, C3M, 06204 Nice, France; jaap.neels@univ-cotedazur.fr; 2 Faculty of Medicine, Côte d’Azur University, 06107 Nice, France; laurain.a@chu-nice.fr (A.L.); esnault.v@chu-nice.fr (V.E.); favre.g@chu-nice.fr (G.F.); 3 Centre National de la Recherche Scientifique, UMR 7073, Laboratory of Physiology and Molecular Medicine (LP2M), 06107 Nice, France; 4 Nephrology, Dialysis and Transplantation Department, University Hospital, 06002 Nice, France; florin.t@chu-nice.fr; 5 Université Côte d’Azur, CHU, 06000 Nice, France; naimi.m@chu-nice.fr; 6 Université Côte d’Azur, CHU, INSERM, C3M, 06000 Nice, France; chevalier.n@chu-nice.fr (N.C.); Giulia.CHINETTI@univ-cotedazur.fr (G.C.)

**Keywords:** macrophages, renin–angiotensin system, diabetic nephropathy

## Abstract

Diabetic nephropathy (DN) is characterized by albuminuria, loss of renal function, renal fibrosis and infiltration of macrophages originating from peripheral monocytes inside kidneys. DN is also associated with intrarenal overactivation of the renin–angiotensin system (RAS), an enzymatic cascade which is expressed and controlled at the cell and/or tissue levels. All members of the RAS are present in the kidneys and most of them are also expressed in monocytes/macrophages. This review focuses on the control of monocyte recruitment and the modulation of macrophage polarization by the RAS in the context of DN. The local RAS favors the adhesion of monocytes on renal endothelial cells and increases the production of monocyte chemotactic protein-1 and of osteopontin in tubular cells, driving monocytes into the kidneys. There, proinflammatory cytokines and the RAS promote the differentiation of macrophages into the M1 proinflammatory phenotype, largely contributing to renal lesions of DN. Finally, resolution of the inflammatory process is associated with a phenotype switch of macrophages into the M2 anti-inflammatory subset, which protects against DN. The pharmacologic interruption of the RAS reduces albuminuria, improves the trajectory of the renal function, decreases macrophage infiltration in the kidneys and promotes the switch of the macrophage phenotype from M1 to M2.

## 1. Introduction

Sustained fasting glycemia over 7.00 mmol/L is the definition of diabetes mellitus that may result either from insulin deficiency following insulin resistance in type 2 diabetes (T2D) or from insulin deficiency caused by destruction of beta cells in the pancreatic islets in type 1 diabetes (T1D). Diabetic nephropathy (DN) is the leading cause of end-stage renal disease in the world and is considered an inflammatory disease. This is illustrated by renal infiltration of monocytes originating from the bone marrow and by lower occurrence of kidney lesions observed in experimental studies targeting disruption of monocytes/macrophages [1]. The inflammatory process is initiated by poor glycemic control and/or high level of albuminuria. Peripheral monocytes enter the kidneys, differentiate into macrophages of diverse phenotypes and their abundancy parallels the renal fibrosis, which negatively correlates with the renal function [2].

Diabetic nephropathy is also characterized by activation of the renin–angiotensin system (RAS). The RAS is an enzymatic cascade which produces various types of angiotensins [3]. At the systemic level, the RAS regulates the arterial tone and the extracellular fluid volume. At the tissue level, the differential expression of the key enzymes and of their receptors may lead to various effects, even to opposite effects [4]. There is a complete RAS intrinsic to the kidneys [5,6] and some components of the RAS intrinsic to the monocyte/macrophage system [7,8]. They both have a dual role. In DN, activated components of the RAS turn out to be harmful: they are proinflammatory, profibrotic and they enhance the oxidative stress. The bulk of experimental data shows that stimulation of the antagonistic components of the RAS may protect against the lesions of DN. In humans, however, only the pharmacological interruptions of the deleterious compounds are efficient [9,10]. In reality, blockade of the activated harmful RAS components in humans decreases blood pressure and albuminuria and improves the trajectory of the renal function [11]. Here, we review the interactions of the kidneys and the monocyte/macrophage system through the RAS in DN in order to highlight the potential treatments available.

## 2. Pathophysiology of DN

### 2.1. Renal Function Consequences of DN

The onset of DN is characterized by the increase in renal size and weight because the tubules and the glomeruli develop hyperplasia and hypertrophy [12]. As a result, the proximal tubule reabsorbs a higher amount of sodium via sodium–glucose cotransporter type 2 (SGLT2), leading to afferent arteriole vasodilation and sustained glomerular hyperfiltration. Glomerular hyperfiltration favors hypertension, renal function loss and albuminuria [13,14]. Albuminuria is measured by the urinary albumin over creatinine ratio (UACR). Microalbuminuria is defined by the UACR from 20 mg/g to 300 mg/g in women and from 30 mg/g to 300 mg/g in men, and macroalbuminuria is defined by the UACR over 300 mg/g. The renal function is characterized by the glomerular filtration rate (GFR), which is used to define hyperfiltration (GFR > 120 mL/min/1.73 m^2^), normal renal function (60 ≤ GFR ≤ 120 mL/min/1.73 m^2^) or renal insufficiency (GFR < 60 mL/min/1.73 m^2^) of varying severity until end-stage renal disease (GFR < 15 mL/min/1.73 m^2^).

In contrast with the description of the five successive stages of DN, which prevailed between the 1980s and the last decade [15], it is now established that microalbuminuria does not necessarily develop into macroalbuminuria and that renal insufficiency occurs and progresses together with micro- or macroalbuminuria. The pathological classification of DN is based on the grade of glomerulosclerosis. The extracellular matrix expansion in the glomeruli starts with the thickening of the basement membrane to end up in the nodular depots inside the glomerular tufts called Kimmerstiel–Wilson nodules, and the pathological classification accepts four classes [16]. However, glomerulosclerosis does not indicate the severity of renal insufficiency, which is only revealed by interstitial fibrosis. It is firmly established that interstitial fibrosis correlates negatively with the renal function in all kinds of nephropathy and most particularly in DN [17,18,19]. The development of renal fibrosis is a complex and multistep process which results from an imbalance between the production and the degradation of the extracellular matrix [20].

### 2.2. Cellular Consequences of Hyperglycemia

Hyperglycemia per se is responsible for kidney cell injuries in proximal tubular cells, mesangial cells and endothelial cells. These cells express glucose transporter 2 (GLUT2) and, as a consequence, they experience a high cytoplasmic glucose level due to diffusion from blood down its chemical gradient. Consequently, the glycolytic pathways provide excess energy supply to the mitochondrial respiratory chain and electrons are transferred to oxygen molecules (O_2_) rather than to mitochondrial transport molecules (cytochromes), resulting in the overproduction of reactive oxygen species (ROS). In turn, ROS reduce glycolysis by inhibiting GAPDH and accumulation of glucose metabolites activates the polyol- and the PKC pathways, thereby favoring production of advanced glycation end-products (AGE) and of N-acetyl glucosamine, leading to renal cell injuries [21]. In addition, increased intraglomerular pressure due to glomerular hyperfiltration promotes albuminuria. Renal production of ROS, glucose metabolites and glycated albumin in urine promote inflammation. Indeed, the release of cytokines and danger-associated molecular patterns (DAMPs) [22] and the production of monocyte chemoattractant protein-1 (MCP-1) in renal cells [23] attract monocytes from the blood into the kidneys; these monocytes then differentiate into macrophages. The presence of macrophages in the kidneys is positively correlated with renal fibrosis in humans [24,25,26].

### 2.3. Experimental In Vivo Models of DN

Hyperglycemia that initiates DN development can be differentially induced according to diabetic animal models. Several experimental models of DN exist, some mimicking the pathological situations observed in T1D and other reconstituting the context of T2D. Indeed, some mice are hyperglycemic due to insulin deficiency, thus recalling the mechanism of T1D. This happens after streptozotocin (STZ) administration that destroys beta cells, mutation of the insulin 2 gene that causes abnormal folding of the protein, resulting in beta cell destruction (AKITA mice), or by genetically driven overproduction of calmodulin in beta cells responsible for beta cell destruction (OVE26 mice). Hyperglycemia may also result from insulin resistance. Mutations in the leptin receptor gene (db/db mice, Zucker diabetic fatty (ZDF) rats and Wistar fatty rats) or in the leptin gene (ob/ob mice) or the overexpression of neuropeptide Y in the hypothalamus in Otsuka Long–Evans Tokushima fatty (OLETF) rats lead to hyperphagia, obesity and insulin resistance, thus mimicking the pathophysiology of T2D [27,28]. STZ treatment can also mimic the metabolic characteristics of T2D in humans when used in combination with a high-fat diet [29] and neonatal administration of STZ is a well-established animal model for T2D in rats [30].

The Animal Models of Diabetic Complications Consortium (AMDCC) defines the key criteria validating a progressive rodent model of DN (https://www.diacomp.org/shared/document.aspx?id=25&docType=Protocol accessed on 28 May 2021): at least 50% decline in GFR, greater than 10-fold increase in albuminuria as compared with appropriate controls and fibrosis measured by mesangial sclerosis, arterial hyalinosis, thickening of the glomerular basement membrane to more than 50% or tubulointerstitial fibrosis [16,31]. Nevertheless, no animal model of DN exhibits all these criteria. For that reason, in the present review, the described models used are specified and not simply referred to as a T1D or T2D model. As in humans, DN in rodents can be accelerated by concomitant induction of hypertension through the knockout of the endothelial nitric oxide synthase (eNOS) gene [32] or through the knock-in of the mouse Ren-2 gene in transgenic (mREN-2)27 rats called Ren-2 rats [33].

## 3. Roles of Monocytes/Macrophages in DN

Tissue-resident macrophages are mostly derived directly from fetal liver or yolk sac during embryogenesis and are described as microglia in the brain, Kupffer cells in the liver and interstitial macrophages in the kidneys. These macrophages are long-lived, do not migrate and are maintained by self-renewal [34]. In contrast, following infection/injury, circulating monocytes originating from the bone marrow migrate into the injured tissue where they differentiate into macrophages [35,36]. These macrophages are called monocyte-derived macrophages or monocyte-derived tissue-resident macrophages and they significantly contribute to diabetes-induced kidney injury. Actually, diverse experimental strategies aimed at impairing monocyte recruitment into the kidneys or reducing their absolute number in the body decrease renal fibrosis. For instance, tubulin inhibition by colchicine alters diapedesis and significantly reduces macrophages and interstitial fibrosis in the kidneys of STZ-treated rats [37]. Furthermore, the whole-body depletion of monocytes/macrophages obtained by intraperitoneal injection of clodronate liposomes [38] or by the administration of diphtheria toxin in the CD11b-DTR model [36] reduces macrophage infiltration and renal fibrosis in STZ-treated mice. CD11b-DTR (diphtheria toxin receptor) mice express the transgene insert that contains a fusion product involving DTR and the green fluorescent protein under the control of the human CD11b promoter [39]. Diphtheria toxin poorly links to murine DTR conferring toxin sensitivity to human DTR specifically expressed in mouse macrophages. These data clearly indicate that renal accumulation of macrophages is a critical factor in the development of DN (Table 1).

### 3.1. Monocyte Recruitment in DN

Monocytes cross the endothelium layer by diapedesis, a multistep process including capture, rolling, slow rolling, arrest, adhesion strengthening, lateral locomotion and monocyte transmigration. Diapedesis involves interactions between endothelial cells expressing ICAM-1 (intracellular adhesion molecule-1) and VCAM-1 (vascular cell adhesion molecule-1) and monocyte ligands such as selectins [62].

In T2D patients, serum ICAM-1 concentration is higher in the presence of microalbuminuria than in patients without microalbuminuria [63]. In the kidneys of db/db mice [58] or ZDF rats [64], ICAM-1 expression is higher than in their non-diabetic counterparts. Icam-1^−/−^ db/db mice [58] or Icam-1^−/−^ STZ-treated mice [46] show a reduced renal macrophage count (Table 1). Further, neutralization of ICAM-1 with a specific monoclonal antibody in STZ-treated mice reduces the number of glomerular macrophages [53]. VCAM-1 is significantly more abundant in the urinary proteome of T2D patients as compared to people without diabetes [65], but the effects of VCAM-1 depletion on the renal macrophage infiltration have not been studied to our knowledge.

Immunohistochemistry analysis of kidney biopsies in humans shows that expression of E- and L-selectins is more abundant in renal vessels from the patients with DN than in vessels from the patients with other kinds of nephropathy. The presence of E-selectin in the peritubular capillaries is positively correlated with the renal macrophage count [66]. In STZ-treated mice, the reduced interaction of L-selectin with its ligands on endothelial cells due to heparan sulfate deficiency significantly reduces the renal macrophage count [47].

The recruitment of monocytes is mainly controlled by chemokines such as MCP-1, also named C–C motif chemokine ligand 2 (CCL2), that binds to C–C chemokine receptor type 2 (CCR2) on the surface of monocytes [67]. Indeed, MCP-1 deletion [40] or blockade by administration of a CCL2-antagonizing L-RNA aptamer [57] or of a CCR2 antagonist [41] decreases macrophage renal infiltration and consequently decreases kidney injury in STZ-treated mice or in db/db mice (Table 1). The synthesis of MCP-1 is under the control of the nuclear factor kappa B (NF kappa B), a transcription factor whose activity is stimulated by tubular reabsorption of excess filtered albumin. NF kappa B controls MCP-1 production in human tubular cells [68] and in the renal cells from uremic rats [69]. In addition, glycated albumin stimulates NF kappa B activity in mesangial cells [70]. In humans, urinary MCP-1 is positively correlated with albuminuria levels [71,72,73] and hyperglycemia according to the level of glycated proteins [70].

Renal infiltration of monocytes also depends on the binding of monocytes to molecules from the extracellular matrix. The renal expression of osteopontin (OPN), a phosphoglycoprotein adhesion molecule, is upregulated in DN in humans, in STZ-treated mice, in db/db mice [74] and in OLETF rats [75]. OPN binds to CD44 on monocytes and promotes monocyte invasion in the kidneys [76]. In STZ-treated hypertensive Ren-2 rats, OPN is overexpressed in mesangial cells [77], podocytes [78], endothelial cells [79] and in tubular cells in association with extensive macrophage accumulation in the kidneys [80,81]. Furthermore, OPN deletion in db/db mice, Akita mice or STZ-treated mice decreases the lesions of DN, indicating that OPN-dependent monocyte recruitment plays an important role in DN [82]. In vitro treatment of human proximal tubular cells by glucose enhances OPN expression, an effect involving toll-like receptor-4 (TLR4) activation [83], phosphatidylinositol 3-kinase- [84] and the beta isoform of protein kinase C [81]-dependent pathways.

Fractalkine (CX3CL1) also drives monocytes into the kidneys since CX3CL1 and its receptor (CX3CR1) are overexpressed in the kidneys of STZ-treated rats, and some CX3CR1^+^ cells are monocytes/macrophages [85]. Presence of renal macrophages decreases in Cx3cr1^−/−^ mice treated with STZ, indicating that their number depends on the CX3CL1/CX3CR1 interaction [42]. In vitro experiments show that CX3CL1 originates from human mesangial cells exposed to AGE [86]. In addition to MCP-1 and CX3CL1, the importance of other chemokines or cytokines in the recruitment of monocytes, such as CXCL8 [43] and IL-17A [44,45], is becoming apparent.

### 3.2. Macrophage Polarization and Plasticity in DN

Once in tissues, infiltrating monocytes differentiate into macrophages according to microenvironmental signals and molecules [87]. Macrophage polarization can be induced in vitro by distinct stimuli and different functional phenotypes are identified based on the expression of several markers (cytokines, growth factors, chemokine receptors and surface antigens) [88,89].

Very schematically, two classes of macrophages are described in in vitro studies. Classically activated (M1) macrophages result from the exposure of monocytes to TH1 cytokines, such as interferon-γ (IFNγ), interleukin (IL) 1β and tumor necrosis factor-α (TNF-α) or to lipopolysaccharides (LPS). M1 macrophages display a high capacity to present antigens and produce high levels of proinflammatory cytokines, such as IL-1β, IL-6, IL-12 and TNF-α, but a low level of IL-10. Chronic M1 macrophage activation can, therefore, mediate ROS-induced tissue damage and impair wound healing. To protect against such tissue damage, the inflammatory response is spatially and temporally counterbalanced by regulatory mechanisms driven by alternatively activated (M2) macrophages. Indeed, macrophages are plastic cells because they can switch from an active M1 to M2 and vice versa upon specific signals. Alternatively activated (M2) macrophages produce anti-inflammatory cytokines such as IL-10, growth factors and profibrotic factors like transforming growth factor-β (TGF-β) involved in the wound healing and fibrosis process. In humans, M2 macrophages express specific markers including CD163, CD206, CD200R, alternative macrophage activation-associated CC-chemokine 1 (AMAC1) [90] and coagulation factor XIII A1 (FXIIIA1). In in vitro experiments, the M2 phenotype can be induced by several combinations of stimuli: TH2 cytokines IL-4 or IL-13 (M2a), immune complexes in combination with IL-1β or LPS (M2b), IL-10 or glucocorticoids (M2c) or by costimulation with TLR ligands and A2 adenosine receptor agonists or by IL-6 (M2d) [91].

M1/M2 classification of macrophages is a simplistic overview of macrophage polarization and functions and does not represent the macrophage phenotypes observed in vivo since the tissue microenvironment is more complex with the simultaneous presence of several stimuli. For instance, M2a, M2b, M2c and M2d are not described in DN, and for that reason, we use here the nomenclature of M1 and M2 macrophages without distinction between the different subsets of the M2 phenotype.

The modulation of macrophage polarization toward the M2 phenotype is associated with a decrease in renal fibrosis as illustrated by several experimental studies (Table 1). Indeed, transfusion of IL-4/IL-13-polarized M2 macrophages in STZ-treated mice protects against tubular atrophy and interstitial fibrosis [49]. Conversely, deletion of cyclooxygenase-2 in STZ-treated mice increases renal M1 macrophages and renal fibrosis [51]. Cellular interactions between macrophages and mesenchymal stem cells increase the ratio of M2/M1 macrophages in the kidneys from STZ-treated mice [38]. Several translational research projects are ongoing in humans to assess the efficacy and safety of intraarterially delivered mesenchymal stem cells/stromal cells from the adipose tissue (NCT03840343) or from the umbilical cord (NCT04562025, NCT04216849) in patients with DN. Low doses of IL-17A reduce the renal macrophage count, IL-6 and TNF-α proteins in urine from db/db mice [44]. Knockout of TLR-2 represses the kidney expression of IL-1β, IL-6, MCP-1, which are M1 macrophage-produced cytokines, in the kidneys of STZ-treated mice [50]. Pentraxin 3 decreases the number of M1 macrophages in the kidneys from STZ-treated mice and promotes the switch toward M2 macrophages, as shown by the induction of arginase 1 and CD206 and by the reduction of inducible nitric oxide synthase (iNOS) and CD16/32 proteins [48]. Hemin, an inducer of the heme oxygenase system, selectively modulates macrophage polarization toward the anti-inflammatory M2 macrophages in the kidneys of ZDF rats or STZ-treated rats [56,61]. In all the aforementioned studies, experimentally induced diabetes is associated with proinflammatory M1 macrophage infiltration in the kidneys. The switch toward the M2 phenotype is associated with the resolution of inflammation and with reduced renal fibrosis, lower albuminuria and/or better renal function.

In humans, the lack of longitudinal studies in the same subjects limits our understanding of macrophage plasticity in DN. In transversal studies, there is a predominance of the M2 phenotype in high pathological grades of DN but the severity of renal fibrosis very likely correlates with the duration and/or with the severity of the proinflammatory phase, which occurred before and was not assessed in these studies. For instance, in an autopsy-based study, the grade of renal fibrosis positively correlated with CD163^+^ M2 macrophages [26] and in a biopsy-based study, the renal M1 macrophage count positively correlated with low fibrosis, whereas the renal M2 macrophage infiltration predominated in patients with high fibrosis [25].

## 4. Regulation of Monocytes/Macrophages by the RAS in DN

### 4.1. Brief Description of the RAS in DN

**Figure 1:**
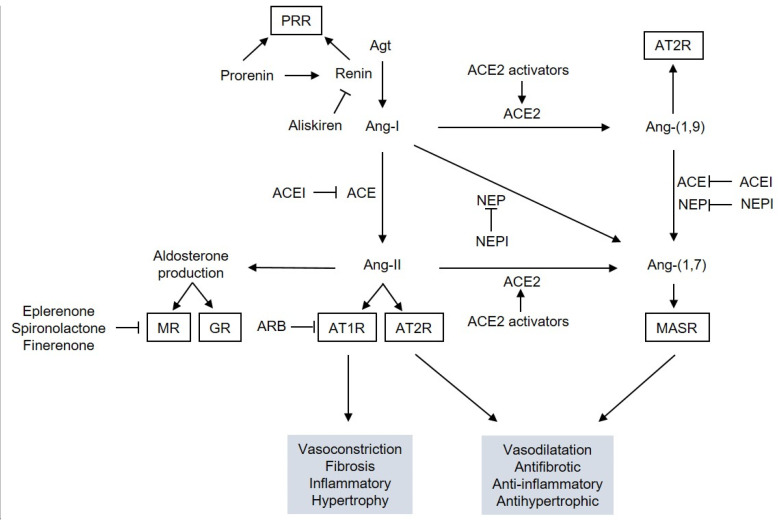
The RAS cascade. Abbreviations: ACE, angiotensin-converting enzyme; ACEI, ACE inhibitor; Agt, angiotensinogen; Ang, angiotensin; ARB, angiotensin receptor blockers; AT1R, Ang-II receptor type 1; AT2R, Ang-II receptor type 2; GR, glucocorticoid receptor; MASR, Mas receptor; MR, mineralocorticoid receptor; NEP, neprilysin; NEPI, NEP inhibitor; PRR, prorenin receptor.

There is the systemic RAS, which regulates extracellular fluid volume and arterial pressure. It contrasts with a local RAS, which is expressed at the tissue level where it warrants non-hemodynamic functions [92]. RAS is an enzymatic cascade (Figure 1). The first and limiting step of the enzymatic cascade is the synthesis of angiotensin I (Ang-I) obtained by cutting off the N-terminal part of angiotensinogen (Agt) by renin. Renin is synthesized as an inactive proenzyme (prorenin) which becomes enzymatically active through either catalytic cutting off the N-terminal propeptide by a convertase, or by a conformational change after its binding to the renin/prorenin receptor (PRR). Besides its enzymatic action, PRR may also trigger intracellular pathways [93]. Ang-I is a ten-amino acid protein that gives rise to several angiotensins. Angiotensin-converting enzyme (ACE) is a dicarboxypeptidase that deletes 2 amino acids at the C-terminal end of several angiotensins. From Ang-I, ACE generates an octopeptide, angiotensin II (Ang-II). From angiotensin 1,9 (Ang-(1,9)), ACE generates angiotensin 1,7 (Ang-(1,7)). ACE type 2 (ACE2) is a monocarboxypeptidase that removes one amino acid at the C-terminal part of Ang-I and Ang-II, generating Ang-(1,9) or Ang-(1,7). Neprilysin (NEP) is a tricarboxypeptidase that deletes three amino acids at the C-terminal end of Ang-I and gives rise to Ang-(1,7) [94]. Ang-II can bind to two G-protein-coupled receptors, Ang-II receptor type 1 (AT1R) and Ang-II receptor type 2 (AT2R). Ang-(1,7) acts on its Mas receptor (MASR). Ang-II and Ang-(1,7) are the main hormones of the RAS. However, the role of other angiotensins is emerging and Ang-(1,9) also exerts direct biological effects in the cardiovascular system by binding to AT2R [95]. Activation of AT1R in the glomerular zone of the adrenal glands induces the synthesis of aldosterone, which acts via the mineralocorticoid receptor (MR) and may also act on the glucocorticoid receptor (GR) with a lower affinity [96].

RAS can be interrupted at several levels (Figure 1). Aliskiren is a direct renin inhibitor which blocks the production of all angiotensins [97]. Angiotensin-converting enzyme inhibitors (ACEI) repress most of the conversion of Ang-I into Ang-II and of Ang-(1,9) into Ang-(1,7). Angiotensin receptor blockers (ARB) target AT1R [11]. Eplerenone, spironolactone [98] and finerenone [99] are MR antagonists (MRA). Thiorphan belongs to inhibitors of NEP (NEPI) [100]. Finally, diminazene aceturate is an activator of ACE2 [100].

### 4.2. Local RAS in the Kidneys

In the kidneys, all members of the RAS are present and regulate renal functions [92]. There is evidence that hyperglycemia favors the production of Ang-II from tubular and glomerular cells [101]. Globally, the Ang-II, ACE, AT1R, MR axis opposes the actions of the Ang-(1,7), ACE2, MASR, AT2R axis. Briefly, the former axis induces matrix expansion, oxidative stress, vasoconstriction and inflammation, whereas the other axis has antifibrotic, anti-inflammatory and vasodilatory effects. The systemic RAS is low in humans suffering from diabetes mellitus, whereas the RAS intrinsic to the kidneys is activated [102]. This paradox might be accounted for by the repression of systemic renin production from the juxtaglomerular apparatus following increased production of Ang-II in glomerular and tubular cells [101,103]. Actually, there is a 100-fold higher level of Ang-II in the tubular fluid and/or in renal homogenates of several animal models of diabetes than in blood [92].

The activation of the RAS intrinsic to the kidneys is highlighted by the fact that ACEI or ARB are important treatments for patients with DN. They lower hypertension or albuminuria [104,105,106] and, remarkably, improve the trajectory of the renal function [107,108]. Blockade of the terminal step of the deleterious axis of the RAS with finerenone (MRA) in addition to ACEI or ARB is even more efficient to prevent the occurrence of a combined endpoint including the decline of the renal function and the occurrence of cardiovascular events than ACEI or ARB alone in patients with DN [109], whereas comprehensive blockade of the RAS cascade with aliskiren added to ACEI or ARB [110] or ACEI on top of ARB [111] does not improve these outcomes.

### 4.3. Local Components of the RAS in Monocytes/Macrophages

Monocytes/macrophages produce Ang-II through the ACE [112] and Ang-(1,7) from Ang-II via ACE2 [113] and express AT1R [114], AT2R [115], MASR [116] and the MR [117]. Furthermore, macrophages from *ldlr*^−/−^ (low-density lipoprotein receptor) mice express Agt and renin in atherosclerotic lesions [118] and PRR was recently detected in human monocyte cell lines, circulating human monocytes and in macrophages infiltrating the kidneys [119,120]. Therefore, it is proposed that the RAS produced in an autocrine manner is essential for monocyte-to-macrophage differentiation and for macrophage functions. Moreover, AT1R regulates the differentiation of bone marrow-derived monocytes into dendritic cells [121].

In pathological conditions, the RAS-dependent differentiation of monocytes into macrophages is disturbed and the two RAS axes oppose their actions. TNF-α downregulates the ACE in human peripheral blood monocytes, thus impairing Ang-II production [122]. AT1R induces oxidative stress in macrophages derived from the human monocytic leukemia cell line THP-1 [114], and in turn, AT1R expression increases in peritoneal macrophages exposed to oxidative stress [113]. In patients on maintenance hemodialysis, losartan (ARB) prevents the development of circulating proinflammatory monocytes [123], suggesting that ARB could regulate the inflammatory status of monocytes in vivo before their recruitment into the inflamed tissues. In patients with atherosclerosis, AT1R favors macrophage infiltration in atherosclerotic lesions as shown by the inhibition of macrophage infiltration in carotid plaques from patients treated with candesartan (ARB) as compared to patients without candesartan [124]. In the murine macrophage Raw 264.7 cell line, Ang-II, irrespective of its receptor, induces the M1 phenotype as measured by the production of the high-mobility group box-1, a DNA damage reparatory protein associated with inflammation [125]. In the same cells, Ang-II promotes macrophage polarization toward the M1 phenotype, also through the connexin 43/NF kappa B signaling [126]. In human primary macrophages, LPS treatment increases the AT1R expression and ARB blocks the secretion of proinflammatory cytokines [124]. In atherosclerosis, the MR seems to promote the M1 macrophage phenotype [117].

MASR is expressed on different subsets of mouse primary macrophages without any difference in expression between unstimulated, LPS/IFNγ-, IL-4/IL-13- and IL-4-polarized macrophages [127,128]. MASR deficiency stimulates the expressions of M1 markers in LPS/IFNγ and inhibits the expression of M2 markers in IL-4/IL-13-treated macrophages. MASR stimulation by Ang-(1,7) decreases the expression of M1 markers in LPS- [129] or LPS/IFNγ-stimulated mouse peritoneal macrophages [128]. Moreover, Ang-(1,7) treatment increases mRNA expression of YM1, an M2 marker, in mouse macrophages stimulated by IL-4 [128]. The anti-inflammatory action of Ang-(1,7) depends on MASR stimulation through inhibition of the TLR4-mediated JNK/FoxO1 signaling pathway in LPS-treated RAW macrophages [130]. MASR knockout increases in vitro migration and T cell activation capacities of peritoneal mouse macrophages [127]. ACE2 overexpression is associated with a significant reduction of Ang-II-induced MCP-1 in THP-1 macrophages [131].

Interestingly, human peripheral blood mononuclear cells exposed to pharmacological doses of renin produce proinflammatory cytokines IL-6, TNF-α and IFNγ independently from the Ang-II–AT1R pathway [119]. Actually, besides its enzymatic role, PRR triggers macrophage infiltration in glomerulonephritis [132] and chronic kidney disease-associated heart failure [133]. More investigations are needed to explore the role of PRR in monocyte/macrophage recruitment and polarization in DN.

Taken as a whole, the ACE, Ang-II, AT1R, MR axis intrinsic to monocytes/macrophages is stimulated in inflammatory conditions and promotes the pro-inflammatory M1 phenotype, whereas the Ang-(1,7), ACE2, MASR axis potentiates the polarization of macrophages into an anti-inflammatory M2 subset.

### 4.4. Modulation of Monocyte Recruitment by the RAS in DN

The ACE, Ang-II, AT1R, MR axis enhances the adhesion of human peripheral monocytes to monolayers of human endothelial cells [134]. P-selectin, E-selectin, ICAM-1 and VCAM-1 expression increases in arterioles and venules of Ang-II-treated rats [135] as well as in aortas of Ang-II-infused rats [136]. Intraperitoneal injection of Ang-II in rats promotes the adhesion of mononuclear cells to arterioles depending on P-selectin and integrin beta 2 [135]. In vitro and in vivo experiments show that Ang-II promotes monocyte adhesion to endothelial cells and migration through ICAM-1 [137]. Since some of these adhesion molecules are upregulated in DN [58,64,65,66], renal production of Ang-II could stimulate their expression in renal endothelial cells to promote migration of monocytes into the kidneys.

Highlighting the role of the RAS intrinsic to the kidneys for monocyte recruitment in DN (Table 2), activation of the ACE and AT1R promotes the accumulation of macrophages in the kidneys of STZ-treated mice through increased MCP-1 expression and NF kappa B activity [138,139]. Renal subcapsular administration of valsartan (ARB) reduces the renal macrophage infiltration in STZ-treated mice [138]. In animal models of DN with hypertension, ACEI decrease the kidney macrophage count. Indeed, in STZ-treated Ren-2 rats, perindopril (ACEI) reduces renal fibrosis [33] and macrophage infiltration [140] depending on OPN [80]. The role of OPN is further documented in OLETF rats treated with ramipril (ACEI) [75]. Similar to the reno-protective effect of ramipril in human patients with DN [106], captopril (ACEI) inhibits renal macrophage infiltration in the kidneys from STZ-treated hypertensive Nos3^−/−^ mice even when blood pressure values are controlled with a diuretic [52]. Further, captopril administration reduces both renal macrophage infiltration and renal fibrosis in db/db mice [141]. Similarly, olmesartan (ARB) decreases interstitial fibrosis and renal macrophage count in STZ-treated rats [142]. Reduction of interstitial fibrosis and decreased expression of the TGF-β protein and of the NF kappa B activity in the kidneys from STZ-treated rats are obtained with telmisartan (ARB) associated with thiorphan (NEPI) or an ACE2 activator (Dize) [100]. More directly, cyclic (c)Ang-(1,7) administration in ob/ob mice decreases the amount of interstitial and glomerular macrophages in the kidneys [143].

Despite the presence of the MR on macrophages [117], its role regarding renal macrophage infiltration or polarization in animal models of DN is not yet documented. However, a 12-week treatment of enalapril (ACEI) in STZ-treated mice or db/db mice does not reduce the expression of the CD11c marker of M1 macrophages in the kidneys. Indeed, CD11c positivity is even higher in enalapril-treated mice than in their untreated counterparts [144]. With regard to intrarenal overproduction of aldosterone in the urine, which is not lowered by enalapril in db/db mice [146], this paradoxical phenomenon may be due to the stimulation of the MR on macrophages. In line with this interpretation, albuminuria is suppressed at week 6 and not any longer at week 12 [144], suggesting that aldosterone might increase albuminuria [147] despite ACE inhibition.

**Figure 2:**
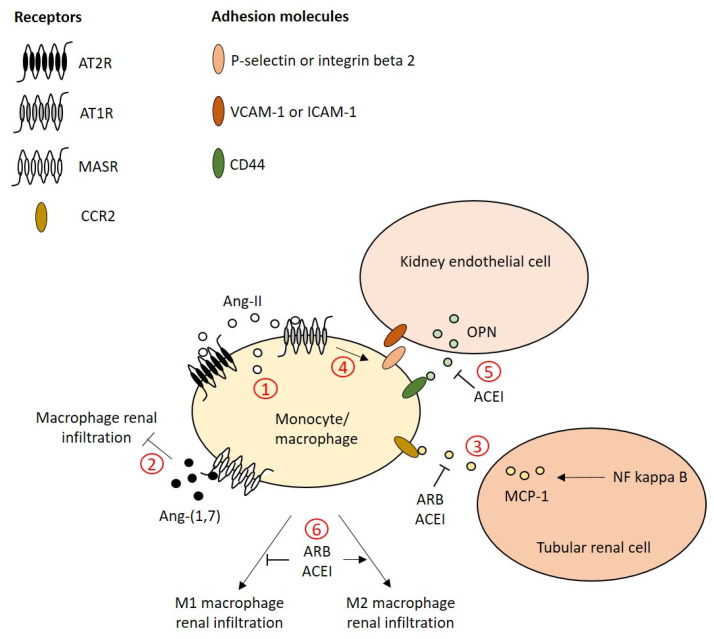
Working model of the RAS-dependent control of monocytes/macrophages in DN. 1. Ang-II is overproduced in the kidneys from diabetic animals and can be released by resident monocytes/macrophages. The RAS system regulates monocyte chemotaxis and recruitment and macrophage polarization in DN. 2. Ang-(1,7) administration inhibits macrophage infiltration in the kidneys. 3. NF kappa B-dependent MCP-1 secretion by tubular renal cells is induced by the Ang-II, AT1R axis. 4. In monocytes, Ang-II stimulates the expression of P-selectin and integrin beta 2 that bind to adhesion molecules, ICAM-1 and VCAM-1, on the surface of endothelial cells. 5. The adhesion of monocytes also involves OPN that is secreted by endothelial cells and then OPN binds to its receptor CD44 on the surface of monocytes. The release of renal OPN depends on the activation of the Ang-II, ACE, AT1R axis. 6. Finally, inhibition of the Ang-II, ACE, AT1R axis in macrophages and/or in the kidney microenvironment induces a switch from the M1 to the M2 macrophage subset alleviating proinflammatory signals and promoting wound healing. Abbreviations: ACEI, angiotensin-converting enzyme inhibitor; Ang, angiotensin; ARB, angiotensin receptor blockers; AT1R, Ang-II receptor type 1; AT2R, Ang-II receptor type 1; CCR2, C–C chemokine receptor type 2; ICAM-1, intracellular adhesion molecule-1; MASR, Mas receptor; MCP-1, monocyte chemoattractant protein-1; NF kappa B, nuclear factor kappa-B; OPN, osteopontin; VCAM-1, vascular cell adhesion molecule-1.

Together, these studies demonstrate that beneficial effects of ACEI or ARB (and maybe of MRA) on renal function, albuminuria and fibrosis are associated with a decreased macrophage count intrinsic to the kidneys (Figure 2). The role of stimulation of the Ang-(1,7), ACE2, MASR axis regarding renal macrophage infiltration remains to be elucidated in DN.

### 4.5. Modulation of Macrophage Polarization by the RAS in DN

The RAS-dependent regulation of macrophage polarization in diabetic kidneys is scarcely documented, but this research field could be more widely studied thanks to technical advances. Indeed, reliable and highly efficient isolation of immune cells from the kidneys has emerged thanks to the development of mechanical tissue disruptors without the collagenase digestion step [148].

Vitamin D treatment, which inhibits the Ang-II, ACE, AT1R, MR axis of the RAS, decreases the number of M1 macrophages, increases the number of M2 macrophages and reduces albuminuria in STZ-treated rats [54]. Losartan induces a phenotype switch in the infiltrated renal macrophages in obese mice following a high-fat diet by increasing M2 markers and decreasing M1 markers [149]. In apolipoprotein E-deficient mice with renal failure induced by the removal of one entire kidney, the administration of bone marrow cells from At1r^−/−^ mice increases the M2 macrophage count in the remaining kidney [150]. Captopril administration in hypertensive eNos^−/−^ db/db mice enhances the expression of M2 markers in the kidneys [141]. Together, these studies suggest that suppression of the Ang-II, ACE, AT1R, MR axis may switch the renal macrophage phenotype from M1 to M2 and contribute to protecting the kidneys from diabetes-related injuries (Figure 2).

**Figure 3:**
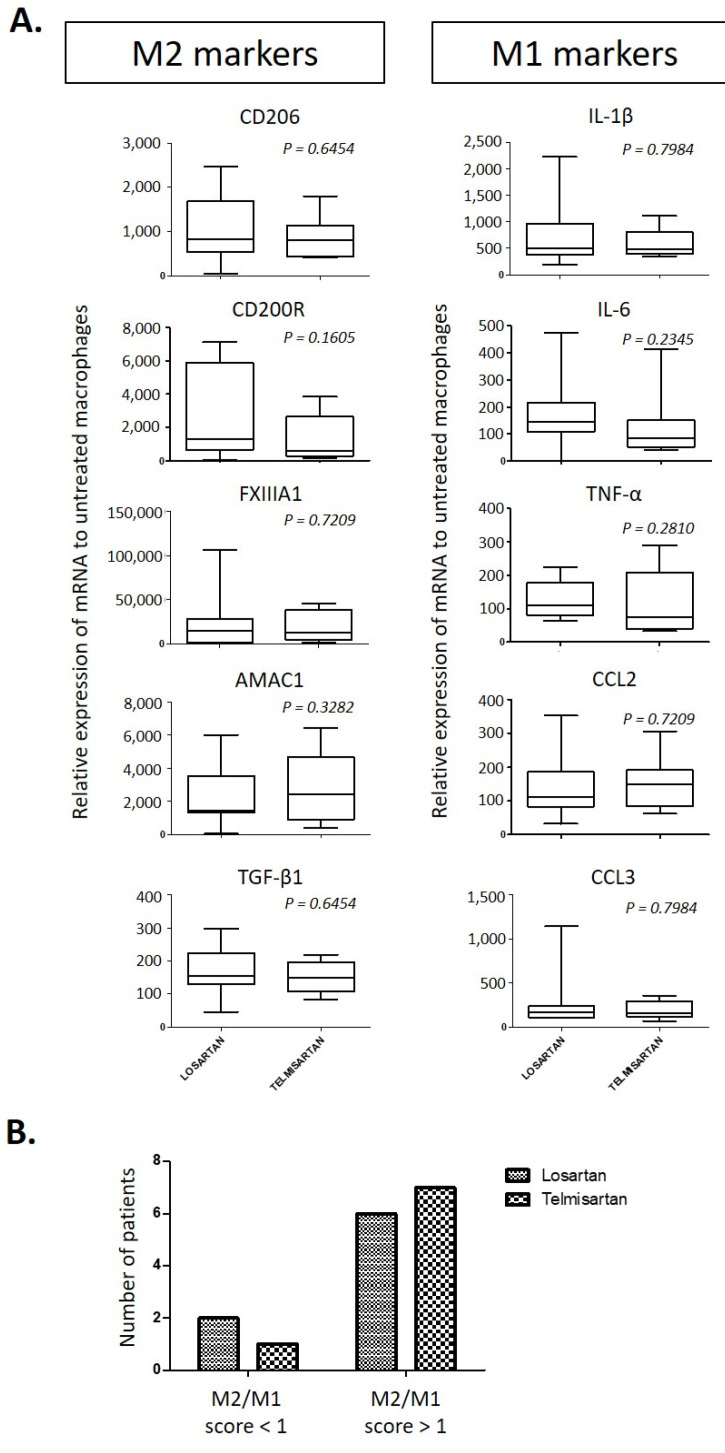
Relative expression of M1 and M2 markers in monocyte-derived macrophages from patients with DN treated with losartan or telmisartan. Blood monocytes isolated from 16 patients treated with losartan or telmisartan (eight per group) for six months were differentiated in vitro into macrophages without cytokines (RM, resting macrophages) or in the presence of 15 ng/mL of IL-1β (M1 macrophages) or IL-4 (M2 macrophages). After six days of differentiation, total RNA was extracted and mRNA expression of M1 (IL-1B, IL-6, CCL2, TNF-α and CCL3) and M2 markers (CD206, CD200R, FXIIIA1, TGF-β1 and AMAC-1) measured by Q-PCR and expressed relative to RM (M2 markers, left panel; M1 markers, right panel). (**A**) The data are presented as the medians (interquartile ranges) and compared using the Mann–Whitney test. The *p*-value between the losartan group versus the telmisartan group is shown. (**B**) Numbers of patients with the M2/M1 score < 1 or > 1 in the telmisartan group and the losartan group. Abbreviations: AMAC1, alternative macrophage activation-associated CC-chemokine 1; CCL2 or CCL3, C–C motif chemokine ligand 2 or 3; FXIIIA1, coagulation factor XIII A1; IL, interleukin; TGF-β1, transforming growth factor beta 1; TNF-α, tumor necrosis factor alpha.

Some data suggest that telmisartan (ARB) could modulate the macrophage phenotype. Indeed, telmisartan represses MCP-1 expression from peripheral monocytes in patients with essential hypertension [151] and drives monocytes to M2 macrophage polarization in mice following stimulation of peroxisome proliferator-activated receptor gamma (PPAR-γ) [152]. Therefore, telmisartan could promote the differentiation of peripheral monocytes toward the M2 macrophage phenotype in patients with DN. To test this hypothesis, a pilot prospective and randomized study was performed at our hospital (NCT02768948). One hundred fifty-four patients with T2D were screened and 24 patients were included with a DN characterized by a GFR > 30 mL/min/1.73 m^2^, micro- or macroalbuminuria without nephrotic proteinuria and hypertension treated with ACEI or ARB. The patients were assigned 80 mg telmisartan or 100 mg losartan daily, as already done in the AMADEO study [153], and they were randomized in order to prevent the confounding effects of various levels of albuminuria on the renal production of MCP-1 since telmisartan is more efficient than losartan in reducing albuminuria [153]. The primary goal was to compare the in vitro polarization potential of circulating monocytes from patients treated with losartan or telmisartan. After six months of treatment, peripheral blood mononuclear cells from these patients were collected to isolate monocytes that were in vitro differentiated into resting macrophages (RM, used as control), M1 macrophages (in the presence of IL-1β) or M2 macrophages (in the presence of IL-4). The secondary objective was to compare the variation of urinary MCP-1 over the creatinine ratio between the losartan and telmisartan groups. Four patients were lost to follow-up, three more patients were not compliant with the study and one was excluded following a rapid decline of the GFR. The data from 16 patients were analyzed: eight patients were treated with losartan and eight patients were treated with telmisartan. The mean age was 68 ± 7 years, the mean GFR was 54 ± 17 mL/min/1.73 m^2^, the body mass index was 29 ± 5 kg/m^2^, the UACR was 342 ± 540 mg/g and HbA1c was 7.3 ± 1.4%. There was no difference in urinary excretion of MCP-1 between the two groups. The fold changes of mRNA expression of M2 or M1 markers (compared to the respective RM) were similar in the two groups (Figure 3A). For each patient, the M2/M1 score was also calculated, corresponding to the ratio of the number of M2 markers to the number of M1 markers that were overexpressed in response to IL-4 or IL-1β, respectively. A score greater than 1 means that macrophages from the patient respond better to stimulation with IL-4 than with IL-1β. Macrophages from 13 patients (six patients with losartan and seven patients with telmisartan) had a score over 1 without differences with respect to losartan and telmisartan administration (Figure 3B). In other words, polarization of macrophages from patients treated with losartan or telmisartan was altered. This specificity of macrophage polarization could depend on DN as well as on the effect of ARB. These results also suggest that ARB therapy could condition macrophages to be less receptive to a deleterious proinflammatory renal environment while retaining their potential to differentiate into the renoprotective M2 phenotype.

## 5. Conclusions

The RAS intrinsic to the kidneys and monocyte/macrophage interactions may worsen the lesions of DN. Consequently, they both offer therapeutic targets to preserve the renal function in patients with DN. Osteopontin, MCP-1 and their respective receptors on monocytes, CD44 and CCR2, are intermediate links between the RAS pathways and monocytes/macrophages. Targeting these molecules in experimental DN limits the recruitment of monocytes into the kidneys and protects the kidneys from diabetes-induced injuries. Some clinical trials targeting leukocyte recruitment with MCP-1 or CCR2 inhibitors or anti-inflammatory molecules are in progress in patients with DN treated with RAS blockers [154].

Suppressing the ACE, Ang-II, AT1R, MR axis of the RAS is the classical treatment of DN, which acts partly by blocking the recruitment of monocytes into the kidneys and by increasing the M2/M1 polarization ratio in kidney-resident macrophages. As M2 macrophages have a kidney-protective role in DN, more experimental work is needed to understand the underlying mechanisms of the modulation of the macrophage phenotype by the RAS in DN. In particular, the effects of the activation of the Ang-(1,7), ACE2, AT2R, MASR axis and the effect of PRR or MR blockade on macrophage polarization have to be investigated in the context of DN. Data from such studies could open new therapeutic avenues to prevent the evolution of DN towards end-stage renal disease.

## Figures and Tables

**Table 1:** Renal effects of strategies targeting monocyte/macrophage recruitment and polarization in animal models of DN.

Diabetic Models	Strategies	Effects on Macrophage Recruitment and Polarization in Kidneys	Renal Effects	Ref.
STZ-treated mice	induced depletion of macrophages with diphtheria toxin	↓ macrophage infiltration	↓ glomerulosclerosis and albuminuria	[36]
	clodronate liposomes		↓ UACR, renal fibrosis and glomerulosclerosis	[38]
	*Mcp-1^−/−^*		↓ albuminuria and renal fibrosis	[40]
	propagermanium (CCR2 antagonist) administration or *Mcp-1^−/−^*		↓ glomerulosclerosis and collagen deposition	[41]
	*Cx3cr1^−/−^*	↓ macrophage infiltration and MCP-1 renal expression	↓ glomerulosclerosis and interstitial fibrosis	[42]
	G31P (antagonist of CXCL8)	↓ macrophage marker expression	↓ glomerulosclerosis and renal fibrosis	[43]
	IL-17A	↓ urinary MCP-1 level and macrophage renal infiltration	↓ glomerulosclerosis	[44]
	*Il-17^−/−^*	↓ macrophage infiltration	↓ albuminuria and glomerulosclerosis	[45]
	IL-17A monoclonal antibodies	no effect on macrophage infiltration	↓ glomerulosclerosis	[45]
	*Icam-1^−/−^*	↓ macrophage infiltration	↓ glomerulosclerosis, ↓ albuminuria and glomerular collagen IV deposition	[46]
	endothelial heparan sulfate deficiency	↓ macrophage infiltration	↓ glomerulosclerosis and interstitial renal fibrosis	[47]
	recombinant pentraxin 3	↓ M1 and ↑ M2 macrophage infiltration	preserved slit diaphragm proteins	[48]
	pentraxin 3 monoclonal antibodies	↑ M1 and ↓ M2 macrophage infiltration	altered slit diaphragm proteins	[48]
	administration of IL-4-/IL-13-treated M2 macrophages	↑ M2 macrophage infiltration	↓ interstitial fibrosis and glomerulosclerosis	[49]
	mesenchymal stem cells	↓ M1 and ↑ M2 macrophage infiltration	↓ UACR, renal fibrosis and glomerulosclerosis	[38]
	*Tlr2^−/−^*	↓ M1 macrophage infiltration, ↓ serum and renal MCP-1 levels	preserved slit diaphragm proteins, normalized renal weight	[50]
	cyclooxygenase-2 deletion in hematopoietic stem cells	↑ macrophage infiltration, ↓ M2 macrophage infiltration and marker expression, ↑ renal MCP-1 expression	↓ deposition of collagen in glomeruli and of α-SMA in interstitium	[51]
STZ-treated mice deficient for Nos3	CCR2 antagonists	↓ macrophage infiltration	↓ UACR and collagen IV deposition in glomeruli	[52]
STZ-treated rats	colchicine	↓ macrophage infiltration, ↓ MCP-1 and ICAM-1 renal expression	↓ albuminuria and ECM accumulation	[37]
	ICAM-1 monoclonal antibodies	↓ macrophage infiltration	correction of glomerular hyperfiltration	[53]
	calcitriol	↓ M1 and ↑ M2 macrophage marker expression	↓ glomerulosclerosis	[54]
	25-OH vitamin D	↓ macrophage infiltration		[55]
	hemin	↓ renal urinary MCP-1 levels, ↓ renal macrophage infiltration, ↓ M1 and ↑ M2 macrophage marker expression	prevented kidney overweight and restored GFR	[56]
db/db mice	CCL2 antagonizing L-RNA aptamer	↓ macrophage infiltration	↓ glomerulosclerosis	[57]
	*Mcp-1^−/−^*		↓ interstitial and glomerular collagen IV deposition, ↓ tubular atrophy	[40]
	IL-17A	↓ urinary MCP-1 level	↓ glomerulosclerosis	[44]
	*Icam-1^−/−^*	↓ macrophage infiltration	↓ glomerulosclerosis, renal fibrosis and albuminuria	[58]
	tectorigenin	↓ macrophage infiltration, ↓ M1 and ↑ M2 macrophage marker expression	preserved slit diaphragm proteins, ↓ glomerulosclerosis	[59]
	*c-fms* monoclonal antibodies	↓ macrophage infiltration, ↓ urine excretion of MCP-1	↓ renal weight without normalization, ↓ hyperfiltration and interstitial collagen deposition	[60]
Ins2Akita mutant mice	IL-17A	↓ urinary MCP-1 level	↓ glomerulosclerosis	[44]
	AMPWAP	↓ M1 and ↑ M2 macrophage marker expression	↓ glomerulosclerosis and albuminuria	[44]
Zucker diabetic fatty rats	hemin	↓ M1 macrophage infiltration and M1 marker expression, ↑ M2 macrophage marker expression	restored GFR, ↓ collagen deposition	[61]

Abbreviations: UACR, urinary albumin-to-creatinine ratio; α-SMA, α-smooth muscle actin; AMWAP, activated microglia/macrophage whey acidic protein; CCR2, C–C chemokine receptor type 2; CXCL8, C–X–C motif chemokine ligand 8; Cx3cr1, CX3C chemokine receptor 3; ECM, extracellular matrix; GFR, glomerular filtration rate; ICAM-1, intracellular adhesion molecule-1; L-RNA, L-ribonucleic acid; MCP-1, monocyte chemoattractant protein-1; Nos3, nitric oxide synthase 3; STZ, streptozotocin; TLR2, toll-like receptor 2.

**Table 2:** Effects of modulation of the RAS on monocyte/macrophage recruitment and polarization in animal models of DN.

Diabetic Models	Strategies	Effects on Macrophage Recruitment and Polarization in Kidneys	Renal Effects	Ref.
STZ-treated mice	enalapril (ACEI)	↑ blood leucocytes and CD68^+^F4/80^+^ cell number, ↑ CD206 (M2 marker) expression in renal macrophages, ↑ fractalkine renal expression	↓ 24-h albuminuria in metabolic cages	[144]
	subcapsular implantation of a valsartan (ARB) delivery sponge in the kidneys	↓ macrophage infiltration	no effect	[138]
STZ-treated hypertensive *Nos3^−/−^* mice	CCR2 antagonist and/or captopril (ACEI)	↓ macrophage infiltration with a CCR2 antagonist, additional effect with captopril	↓ UACR with CCR2 antagonist and collagen IV deposition in glomeruli, no additional effect with captopril	[52]
STZ-treated rats	olmesartan (ARB)	↓ macrophage infiltration	↓ glomerulosclerosis, interstitial fibrosis	[142]
	losartan (ARB) and/or mycophenolate mofetil (macrophage infiltration and proliferation suppressor)	↓ macrophage infiltration and MCP-1 renal expression, additional effect with mycophenolate mofetil, no effect on ICAM-1 expression	↓ kidney weight and glomerulosclerosis, additional effect with mycophenolate mofetil	[145]
	candesartan (ARB) or enalapril (ACEI)	↓ MCP-1 renal expression and macrophage infiltration	↓ kidney weight	[139]
	thiorphan (NEPI) or diminazene aceturate (ACE2 activator) and telmisartan (ARB)	not available	↓ glomerular and tubulointerstitial fibrosis	[100]
STZ-treated hypertensive REN-2 rats	no treatment	not available	severe glomerulosclerosis, low GFR	[33]
	perindopril (ACEI)	↓ macrophage infiltration	↓ renal fibrosis and protection against GFR decrease	[140]
db/db mice	enalapril (ACEI)	↑ blood leucocyte and macrophage number, ↑ CD11c (M1 marker) expression in renal macrophages	↓ 24-h albuminuria in metabolic cages	[144]
ob/ob mice	cAng-(1,7) and/or lisinopril (ACEI)	↓ macrophage infiltration, additional effect with lisinopril	↓ glomerulosclerosis, albuminuria, renal fibrosis, additional effect with lisinopril	[143]
*eNos^−/−^* and db/db mice	captopril (ACEI)	↓ macrophage infiltration, ↑ arginase-1 and IL4-RA (M2 markers) expression	↓ UACR, glomerulosclerosis and interstitial fibrosis	[141]
Otsuka Long-Evans Tokushima fatty rats	ramipril (ACEI)	↓ macrophage infiltration and osteopontin expression	↓ glomerulosclerosis and tubulointerstitial fibrosis	[75]

Abbreviations: ACE2, angiotensin-converting enzyme type 2; ACEI, angiotensin-converting enzyme inhibitors; ARB, angiotensin II receptor blockers; cAng-(1,7), cyclic angiotensin 1,7; CCR2, C–C chemokine receptor type 2; eNOS, endothelial nitric oxide synthase; GFR, glomerular filtration rate; ICAM-1, intracellular adhesion molecule-1; IL4-RA, interleukin 4 receptor alpha; MCP-1, monocyte chemoattractant protein-1; NEPI, neprilysin inhibitors; Nos3, nitric oxide synthase 3; STZ, streptozotocin; UACR, urinary albumin-to-creatinine ratio.
